# Facility-Based Yoga for Adult Cancer Patients and Survivors at a National Cancer Institute (NCI)-Designated Comprehensive Cancer Center: A Retrospective Study of Program Utilization and Barriers to Care

**DOI:** 10.3390/healthcare14213830

**Published:** 2026-11-09

**Authors:** Maria Grosso Zelaya, Margo Brooke Gerke, Reza Lahiji, Gloria Fung, Moshelle Borjigin, Sejla Zahirovic, Hanna Slomik, Joseph Palese, Anna Bausum, Jennifer S. Mascaro, Almira Catic, Carla Haack, Neil M. Iyengar, Kevin Kalinsky, Viraj A. Master

**Affiliations:** 1 Integrative Oncology and Survivorship, Winship Cancer Institute, Emory University, Atlanta, GA 30322, USA; 2 Emory University School of Medicine, Atlanta, GA 30322, USA; 3 Department of Urology, Emory University School of Medicine, Atlanta, GA 30322, USA; 4 Department of General Surgery, Emory University School of Medicine, Atlanta, GA 30322, USA; 5 Department of Family and Preventive Medicine, Emory University School of Medicine, Atlanta, GA 30322, USA; 6 Department of Hematology and Medical Oncology, Emory University School of Medicine, Atlanta, GA 30322, USA

**Keywords:** cancer, integrative oncology, yoga therapy, patient-reported outcomes, feasibility

## Abstract

Background: Yoga is associated with improvements in quality of life and symptom burden in patients with cancer, yet barriers to participation persist. This study retrospectively evaluated real-world utilization, factors associated with attendance, symptom trajectories, and satisfaction with a yoga program at an NCI-designated comprehensive cancer center. Methods: Adults with a primary cancer diagnosis enrolled in the Integrative Oncology and Survivorship Program (January 2024–February 2026) who attended ≥1 yoga session were included. Patients attended weekly 60 min instructor-led classes. Attendance trends, baseline predictors of attendance, pre/post PROMIS-29 changes after four visits, and satisfaction ratings were analyzed using chi-square, rank-sum, and multivariable negative binomial regression. Results: Among 148 patients, 80 (54.1%) attended ≥4 visits. Median age was 60 years; 44.6% identified as Black/African American. Attendance increased over time (70.1% in 2024 to 75.0% in early 2026). Patients aged ≥60 demonstrated 1.74-fold (IRR 1.74, 95%CI 1.05–2.90, *p* = 0.033)-higher attendance, while mild sleep disturbance was associated with lower attendance (IRR 0.26, 95%CI 0.09–0.74, *p* = 0.012). No significant PROMIS-29 domain changes were observed after four visits, though descriptive improvements were noted. Satisfaction was uniformly high; 93.8% rated likelihood of recommending the service as “Very Good”. Conclusions: In this retrospective real-world study, facility-based yoga was well accepted and demonstrated improving utilization across a racially diverse oncology cohort. Older age was associated with greater engagement, while sleep disturbances may represent a barrier to sustained participation. and demonstrated improving utilization across a racially diverse oncology cohort. Older age was associated with greater engagement, while sleep disturbances may represent a barrier to sustained participation.

## 1. Introduction

Yoga-based interventions have demonstrated efficacy in several domains including improvements in quality of life, fatigue, sleep, stress, social function, anxiety, and depression among patients with and survivors of cancer [1,2,3,4]. As such, yoga-based integrative approaches are being increasingly utilized in the US by cancer patients/survivors, with an estimated 16% of patients participating in yoga-based practices in 2025 [5,6]. Yoga therapy for cancer survivors has rapidly expanded, and yoga is offered at more than 85% of NCI-designated Comprehensive Cancer Centers [7]. Furthermore, the American Society of Clinical Oncology (ASCO) now recommends yoga for reducing anxiety/depression and enhancing the quality of life of patients with cancer [8].

Despite the documented benefits and growth of yoga practices, yoga remains underutilized among cancer patients and survivors. Research on the routine integration of yoga into cancer care has been disproportionally concentrated in certain populations, with non-Hispanic White patients being the predominant racial group represented in the current literature [9]. However, despite the predominance of White patients in existing studies, interest in yoga practice has been shown to be higher among non-White patients, females, employed patients, and those with a more recent cancer diagnosis, suggesting disparities in access for some patient populations interested in yoga [10]. As such, there is a need for greater representation of diverse racial and ethnic populations in research investigating yoga-based interventions.

Patient-reported barriers to yoga utilization have included insufficient time, motivation difficulties, symptom burden, and poor physical balance [10]. Additionally, lack of awareness of both the availability and benefits of yoga have also been documented as two common barriers to engagement [10,11,12]. In populations in which yoga has been less studied, namely older adult males from racially diverse backgrounds, the most commonly identified barriers to yoga therapy included lack of knowledge or skepticism of yoga, disinterest, physical health limitations, and logistical barriers such as transportation, affordability, and time [12]. Among veteran populations, baseline interest in yoga was low with approximately 78% of patients reporting no interest in yoga; however, this lack of interest decreased to 46.4% after a brief educational script [12]. Thus, understanding real-world utilization and the factors associated with sustained attendance is essential to identifying barriers to engagement and tailoring yoga services to meet patients where they are.

With the demonstrated utility of yoga among cancer patients and survivors, there is a need for further research on the implementation of such practices, symptom trajectories, and patient satisfaction. This study analyzed a cohort of adult cancer patients and survivors in a racially diverse population who expressed interest in participating in a free-of-cost yoga service. Its primary aims were fourfold, (1) to establish yoga attendance rates over our study period, (2) to identify factors associated with yoga attendance, (3) to characterize symptom burden changes associated with yoga attendance, and (4) to establish patient satisfaction with the yoga services offered.

## 2. Methods

### 2.1. Patient Inclusion Criteria and Characteristics

Established patients seen by the Integrative Oncology and Survivorship (IOS) Program at the Winship Cancer Institute from January 2024 to February 2026 were included in this study. Eligibility criteria were defined as being ≥18 years, having a primary cancer diagnosis, and completing at least one session of yoga. All services offered by the Integrative Oncology and Survivorship Program are free of cost and subsidized by internal grants and external donors. Patients enrolled in the yoga service are not charged for any of the sessions. Due to this, their health insurance status and coverage are not reviewed by the team and scheduling staff.

Patients are initially seen by a naturopathic physician in an individual consult and/or a group medical visit (GMV). During the session, they explain different integrative services and therapies offered by the IOS Program, including yoga. Clinicians ask clarifying questions about patient mobility and movement capacity to ensure that patients can safely and healthily perform the exercises taught by the instructor. Patients who are explicitly interested in yoga are referred to the service and are called for scheduling by team members afterward. Study recruitment was based on convenience sampling. Patients are initially seen by a naturopathic physician in an individual consult and/or a group medical visit (GMV). During the session, they explain different integrative services and therapies offered by the IOS Program, including yoga. Clinicians ask clarifying questions about patient mobility and movement capacity to ensure that patients can safely and healthily perform the exercises taught by the instructor. Patients who are explicitly interested in yoga are referred to the service and are called for scheduling by team members afterward. Study recruitment was based on convenience sampling.

Characteristics collected from electronic medical records included age, gender, race, body mass index (BMI, kg/m^2^), Charlson Comorbidity Index (CCI), Eastern Cooperative Oncology Group (ECOG) performance status, primary cancer diagnosis, cancer status, and number of yoga sessions attended. Any patients with incomplete PROMIS-29 data were excluded from pre-to-post symptom improvement analyses. The Emory University IRB determined that this project does not involve human subject research or clinical investigation; therefore, it does not require full IRB approval or informed consent. All protected health information was gathered and de-identified in compliance with standard clinical protocols prior to data extraction.

### 2.2. Oncology Yoga Service Details

The Winship Cancer Institute’s yoga service is organized as a 60 min class every Monday at 9:30 am and 10:45 am in Midtown Atlanta. Each class is limited to 12 participants and led by a licensed oncology yoga instructor. The oncology yoga instructor is a registered yoga therapist (RYT) with over 1000 h of experience. Participants are initially scheduled for four visits to assess tolerance, interest, and feasibility [13], after which they may continue attending without a predefined limit. Each session incorporates breath regulation, gentle postural sequences, supported stretching, light strengthening, and guided relaxation. Sessions typically begin with breathwork to promote relaxation and awareness, followed by restorative and mobility-focused exercises, brief strengthening activities targeting major muscle groups, and conclude with guided relaxation emphasizing diaphragmatic breathing. Programming is individualized within a group setting to accommodate heterogeneous cancer diagnoses, treatment phases, and functional abilities. Yoga props, including mats, bolsters or pillows, foam blocks, blankets, and soft straps, are provided to enhance safety and support. All sessions are free of cost.

### 2.3. PROMIS-29 Questionnaire and Outcome Measures

Questionnaire responses were collected from patients at baseline and after completion of the 4-visit regimen. The PROMIS-29 v2.0 measures changes in functioning and wellbeing by assessing seven physical, social, and psychological symptom domains; each is represented by four items and uses a 5-point Likert scale [13]. Standardized scores are calculated and utilized to categorize patients into Normal, Mild, or Moderate/Severe per domain [13]. The Physical Function and Social Participation domains are positively scored; an increase in T-scores signals an improvement in the symptom domain. The remaining five domains are negatively scored. The PROMIS-29 tool has been extensively validated in oncology populations and is widely used in behavioral and supportive care research [14].

The terms “engagement/attendance” are used interchangeably and were defined as the number of scheduled sessions that were subsequently attended by patients. Attendance rates were calculated by dividing the total number of scheduled appointments by the total number of attended appointments. Patients who cancel or no-show for the week are marked as absent or not present. Both monthly and yearly yoga engagement were evaluated to account for observation window variability. Patient satisfaction was evaluated using a post-session survey administered to new yoga participants after their first visit (Supplemental File S1). The survey was internally created by the physicians and clinical research team to assess service feasibility. It consisted of six items evaluating the instructor’s friendliness, concern, attentiveness, and expertise, as well as the likelihood of recommending yoga to others. Each item was rated on a 5-point Likert scale where 1 was “Very Poor” and 5 was “Very Good.” A final open-ended question allowed patients to elaborate on their experience in their own words; this question was excluded from analysis due to limited completion rates.

### 2.4. Data Analysis

Categorical variables were reported as total count with percentages, while continuous variables were documented as median values with interquartile ranges (IQR). To analyze differences between yoga attendance groups (i.e., <4 vs. ≥4 sessions), Chi-square tests of association were run for categorical variables and rank sum tests for continuous variables. Multivariable negative binomial regression models then analyzed factors associated with increasing yoga attendance. The first model analyzed sociodemographic factors associated with yoga attendance, and the second analyzed PROMIS-29 domains associated with yoga attendance, with two separate models run to prevent overfitting. Negative binomial regression was used to adjust for potential right-skew and overdispersion of data [15]. Incidence rate ratios (IRRs) with 95% confidence intervals (CI) were then reported. To evaluate improvement in symptom burden following the four visits, rank-sum tests were run to assess median pre-post changes in PROMIS-29 domain T-scores among those who completed the initial yoga session course and PROMIS-29 questionnaires. Yoga engagement metrics and patient satisfaction ratings were descriptively analyzed. A significance threshold of *p* < 0.05 and two-tailed tests were used for the purposes of this study. Analyses were conducted using STATA, version 19.5 (StataCorpLLC, College Station, TX).

## 3. Results

148 patients met inclusion criteria. No formal a priori sample size calculation was performed, as the study included all eligible patients during the study period. 80 (54.1%) attended 4 or more yoga visits and 143 (96.6%) completed the PROMIS-29 questionnaire at baseline. 117 (79.1%) patients were female with a median (IQR) age of 60 (52–68) years. 75 (50.7%) patients were White and 66 (44.6%) were Black/African American. When evaluating PROMIS-29 domains, depression was the best-rated domain with 106 (74.1%) patients reporting normal symptomatology. Physical function was the lowest rated domain with 82 (57.3%) patients reporting Mild, Moderate, or Severe symptomatology. Total cohort demographic and clinical features, alongside baseline PROMIS-29 characteristics, are illustrated in Table 1. Primary diagnoses were varied, with 87 (58.8%) patients diagnosed with breast cancer, 13 (8.8%) with prostate cancer, and the remainder of the cohort diagnosed with various other malignancies depicted in Supplemental Table S1.

### 3.1. Yoga Engagement and Attendance Trends

**Figure 1:**
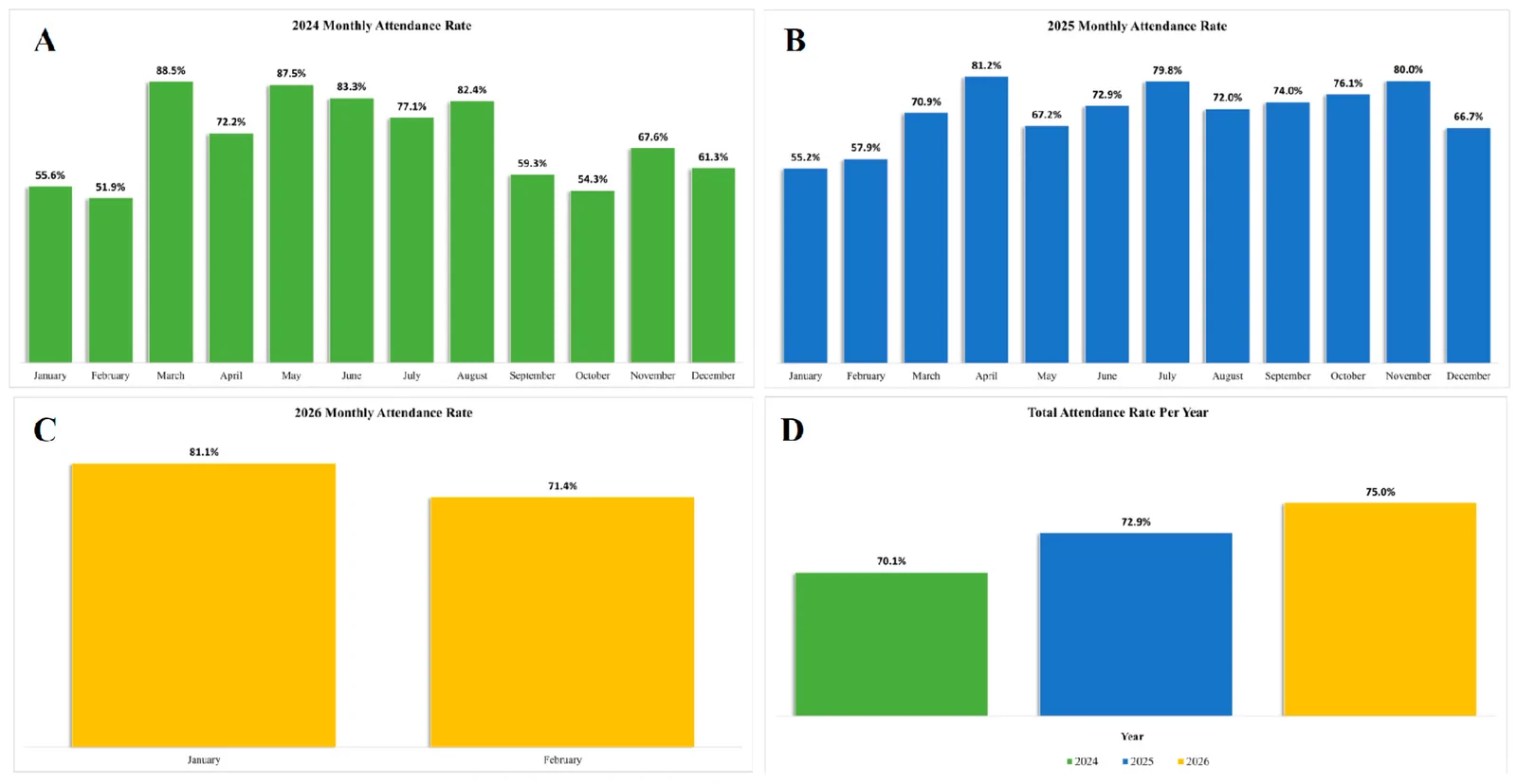
Monthly and yearly yoga attendance rates. (**A**) Monthly attendance rates in 2024. (**B**) Monthly attendance rates in 2025. (**C**) Monthly attendance rates in 2026. (**D**) Yearly attendance rates across the study period (2024–2026).

To gauge engagement with the service, visit attendance was captured. A modest upward trend over time is shown, as overall attendance increased from 70.1% (Mdn 69.9%, IQR 57.5–82.9%) in 2024 to 72.9% (Mdn 72.5%, IQR 67.0–78.0%) in 2025 to 75.0% in the early 2026 period. Specific month-by-month breakdown of attendance rates are illustrated in Figure 1A–C. Total attendance rates per year were calculated and visualized in Figure 1D. Across all calendar years, attendance demonstrated by-month variability yet with an overall increasing average rate by-year.

### 3.2. Factors Associated with Longer Yoga Attendance

Table 2 presents patient factors stratified by yoga attendance groups. Those attending ≥4 visits were significantly older, with a greater proportion of patients aged 60 years or older (63.8% vs. 35.2%; *p* = 0.001) and a higher median age (63.5 vs. 56.0 years; *p* < 0.001). No significant differences were observed by gender, race, BMI, cancer status, Charlson Comorbidity Index severity, or ECOG performance status between attendance groups. Baseline PROMIS-29 classifications were similar across groups, with sleep disturbance being the only domain demonstrating a statistically significant difference. Patients attending ≥4 visits reported a higher proportion of Normal sleep symptomatology (78.9% vs. 58.2%; *p* <0.001).

Negative binomial models evaluating factors associated with greater yoga attendance are as follows. With regards to sociodemographic factors, patients aged ≥ 60 years had a 1.74-fold (IRR 1.74, 95%CI 1.05–2.90, *p* = 0.033) higher rate of yoga attendance compared to those aged <60, with no other variables associated with yoga attendance (*p* > 0.05). Sociodemographic associations with yoga attendance are depicted in Table 3. When analyzing PROMIS-29 domains associated with attendance, patients with a mild sleep disturbance demonstrated 0.26-fold (IRR 0.26, 95%CI 0.09–0.74, *p* = 0.012) the rate of yoga attendance compared to patients without any sleep disturbance. PROMIS-29 associations with yoga attendance are depicted in Table 4.

### 3.3. Pre-to-Post Yoga Symptom Burden Changes

Table 5 represents PROMIS-29 symptomatology distributions and median T-scores at baseline and post-yoga among patients who attended ≥4 visits (*n* = 62). No statistically significant changes were observed across any PROMIS-29 domain from pre-to-post intervention assessment. Classification levels remained largely stable across time, with some minor improvement in median domain T-scores.

### 3.4. Patient Satisfaction with Services Received

Overall patient satisfaction ratings were uniformly high across all items, demonstrating minimal variability. A mean rating of 4.98 out of 5 was provided by respondents when asked if they would recommend the yoga service to others (Question 6), with 93.8% of patients selecting the “Very Good” category.

## 4. Discussion

This retrospective study conducted at an NCI-designated Comprehensive Cancer Center utilizing real-world data demonstrated that a yoga service is feasible and acceptable among racially diverse patients with cancer. Key findings included improving trends in yoga utilization over time, identification of key factors associated with yoga attendance/non-attendance, and excellent patient satisfaction. Attendance rates and general patient acceptability were high, in comparison to similar pilot yoga programs instituted at other national comprehensive cancer centers [16]. Pending the validation of our results, these findings could aid in optimizing scheduling protocols, improving attendance rates, and may foster a greater understanding of yoga services at both the patient and department level. More broadly, this program may serve as a model for integrating yoga into routine care at other cancer centers, consistent with ASCO-endorsed guidelines recommending yoga for improved quality of life. Wider adoption of no-cost, facility-based yoga services may help reduce disparities in access to evidence-based integrative therapies, particularly for patients historically underrepresented in yoga research.

In our cohort, 49% of patients were non-White, including 44.6% who identified as Black or African American. As such, this study is distinguished by one of the highest reported percentages of patients who self-identified as Black or African American, including in comparison with large-scale clinical trials specifically targeting ethnic populations [16]. Historically, research in yoga therapy for cancer patients has lacked racial diversity, with meta-analyses frequently not reporting race/ethnicity [16]. The large representation of Black or African American patients in our single-center cohort also outpaces the general population (12.6%) of US adults who reported yoga usage as of 2022 [16]. Importantly, however, yoga therapy may be particularly important for non-White patients with cancer who have consistently experienced poor mental health-related quality of life (QoL) outcomes compared with their White counterparts [17].

Prior research has demonstrated a correlation between race and the interest but non-use of yoga [17]. Non-White survivors, particularly Black or African American, want to participate, but may not have easy access to yoga services nationwide due to geographical and QoL barriers. The central location of our cancer institute in midtown Atlanta could aid in widening accessibility, as well as the removal of cost. Despite our program engaging a substantially more racially diverse population than prior studies, no significant differences in visit completion rates were observed by race which may reflect scheduling optimization and department outreach efforts.

Furthermore, this study found that patients aged 60 and older were significantly more likely to attend yoga classes, suggesting greater interest among older patients than represented in the current literature [18,19]. Our classes were offered on Mondays at 9:30am and 10:30am, which may better accommodate retired individuals with more flexible schedules and fewer work-related scheduling constraints [20]. These patterns may inform program design to broaden access. Offering additional session times, such as evenings, weekends, or virtual classes, may better accommodate younger patients with work or caregiving obligations. Additionally, maintaining free, centrally located services may help sustain engagement among racially diverse patients who face cost and access barriers. Aligning yoga sessions with scheduled clinic appointments may further reduce transportation and time burdens for patients in active treatment. Future studies that assess patient-reported barriers to participation may help better tailor program delivery to meet diverse needs.

Aside from mild sleep disturbance, no other patient characteristics were significantly associated with yoga attendance. Patients with mild sleep disturbances were found to attend yoga sessions less frequently compared to their normal counterparts. This finding is likely multifaceted, with disease and treatment related factors likely contributing to sleep disturbances and thus the reduced attendance rates observed. This includes active treatment vs. survivorship, different types of endocrine therapies and chemotherapy agents, use of medication for sleep disturbance or other symptoms [21,22,23]. While patients with Moderate/”Severe” sleep disturbances did not demonstrate such an association, it may be that these patients are paradoxically more motivated to seek yoga/integrative therapies for potentially severe concurrent symptoms [24]. Patients with mild sleep disturbance may not take pharmacological treatments to manage symptom concerns and seek to utilize yoga as a remedy. Further research is required to validate such claims, and this finding remains hypothesis-generating.

Additionally, the general lack of symptom improvement could be influenced by yoga frequency. Patients participate only once a week for 60 min, which may be insufficient to significantly manage complex symptoms like depression or physical function. Previous studies have shown significant reduction in symptom interference when patients attended yoga multiple times a week and for more than four sessions [24]. Future program evaluation could examine different frequency and duration of the yoga service to identify optimal windows to improve patient symptoms.

This study’s findings should be contextualized in light of several limitations. First, this study’s retrospective, feasibility-based design and lack of control arm limits clinical interpretation and causality. Second, as a single-center study, generalizability is limited. Additionally, given that referral to the yoga program required patients to express prior interest in participating, our findings primarily reflect engagement and satisfaction rates among a motivated subgroup and may not be generalizable to the broader oncology population. Additionally, as participation required travel to an in-person class and clinician screening for mobility, these findings may not generalize to patients receiving home-based palliative or hospice care. Third, despite our desire to limit this by having one trained instructor run all classes, due to the nature of yoga there is substantial heterogeneity in the practice potentially confounding symptom-related results. Fourth, detailed disease and treatment-related information was not collected or analyzed, likely confounding our results and limiting our ability to account for differences in disease burden, treatment-related needs, and functional status that may influence yoga participation. Fifth, our modest sample size may have limited the power of our analyses precluding significance, particularly when analyzing pre-and post-yoga PROMIS-29 score distributions where only 62 (41.9%) patients were included. Sixth, the satisfaction survey is not a psychometrically validated instrument, but an internally created tool to preliminary examine patient acceptance of the program. Because this survey was administrated after the initial session, high satisfaction may be due to novelty bias, rather than true yoga service satisfaction. Future prospective multicenter studies are encouraged to establish the efficacy of yoga and to further optimize their integration into current clinical workflows. as participation required travel to an in-person class and clinician screening for mobility, these findings may not generalize to patients receiving home-based palliative or hospice care. Third, despite our desire to limit this by having one trained instructor run all classes, due to the nature of yoga there is substantial heterogeneity in the practice potentially confounding symptom-related results. Fourth, detailed disease and treatment-related information was not collected or analyzed, likely confounding our results and limiting our ability to account for differences in disease burden, treatment-related needs, and functional status that may influence yoga participation. Fifth, our modest sample size may have limited the power of our analyses precluding significance, particularly when analyzing pre-and post-yoga PROMIS-29 score distributions where only 62 (41.9%) patients were included. Sixth, the satisfaction survey is not psychometrically validated instrument, but an internally created tool to preliminary examine patient acceptance of the program. Because this survey was administrated after the initial session, high satisfaction may be due to novelty bias, rather than true yoga service satisfaction. Future prospective multicenter studies are encouraged to establish the efficacy of yoga and to further optimize their integration into current clinical workflows.

## 5. Conclusions

In this retrospective study at an NCI-designated comprehensive cancer center, a free, facility-based yoga program was feasible and well accepted among a racially diverse cohort of cancer patients and survivors, with attendance improving over the study period. Older age was associated with increased attendance rates, in contrast to patients with sleep disturbances who attended yoga sessions less frequently. Symptom burden did not change significantly after four sessions. Future prospective studies should evaluate whether greater session frequency or longer program duration improves symptom outcomes, and whether addressing sleep-related barriers increases participation. No generative AI tools were used in the development of this manuscript or content therein.

## Figures and Tables

**Table 1:** Study cohort characteristics and baseline PROMIS-29 questionnaire results.

Covariate	Subgroup	Total (n = 148)
Age (years)	<60	73 (49.3%)
	≥60	75 (50.7%)
	Median (IQR)	60 (52.5–68.0)
Gender	Female	117 (79.1%)
	Male	31 (20.9%)
Race	Non-White	73 (49.3%)
	White	75 (50.7%)
	American Indian or Alaska Native	1 (0.7%)
	Asian	3 (2%)
	Black or African American	66 (44.6%)
	Other	3 (2%)
Cancer Status	Survivorship	88 (59.5%)
	Active	60 (40.5%)
BMI (kg/m^2^)	Non-obese (<30)	107 (72.3%)
	Obese (≤30)	41 (27.7%)
	Median (IQR)	26.5 (24.2–30.6)
Charlson Comorbidity Index	Non-severe	82 (55.4%)
	Severe	66 (44.6%)
	Median (IQR)	4 (3–6)
ECOG Performance Status	ECOG 0	107 (72.3%)
	ECOG 1+	41 (27.7%)
	Median (IQR)	0 (0–1)
Number of visits	<4 Visits	68 (45.9%)
	≥4 Visits	80 (54.1%)
	Median (IQR)	4 (2–7)
Physical Function Domain *	Normal	61 (42.7%)
	Mild	44 (30.8%)
	Moderate/Severe	38 (26.5%)
	Median (IQR)	43.2 (38.9–56.9)
Anxiety Domain *	Normal	89 (62.2%)
	Mild	29 (20.3%)
	Moderate/Severe	25 (17.5%)
	Median (IQR)	51.7 (40.3–57.8)
Depression Domain *	Normal	106 (74.1%)
	Mild	25 (17.5%)
	Moderate/Severe	12 (8.4%)
	Median (IQR)	48.9 (41.0–55.5)
Fatigue Domain *	Normal	83 (58.0%)
	Mild	28 (19.6%)
	Moderate/Severe	32 (22.4%)
	Median (IQR)	51 (46.2–58.9)
Sleep Disturbance Domain *	Normal	99 (69.2%)
	Mild	23 (16.1%)
	Moderate/Severe	21 (14.7%)
	Median (IQR)	51.7 (46.4–57.4)
Social Participation Domain *	Normal	86 (60.1%)
	Mild	38 (26.6%)
	Moderate/Severe	19 (13.3%)
	Median (IQR)	49.7 (44.2–53.6)
Pain Interference Domain *	Normal	65 (45.5%)
	Mild	37 (25.9%)
	Moderate/Severe	41 (28.6%)
	Median (IQR)	55.7 (41.6–60.2)

* 143 out of 148 patients completed a PROMIS-29 questionnaire. Abbreviations: Interquartile range (IQR).

**Table 2:** Cohort characteristics and baseline PROMIS-29 scores by 4-visit regimen attendance.

Covariate	Subgroup	<4 Visits (n = 68)	≥4 Visits (n = 80)	*p*-Value
Age (years)	<60	44 (64.7%)	29 (36.3%)	**0.001**
	≥60	24 (35.2%)	51 (63.8%)	
	Median (IQR)	56 (50.0–64.5)	63.5 (55.5–70.5)	**<0.001**
Gender	Female	58 (85.3%)	59 (73.8%)	0.085
	Male	10 (14.7%)	21 (26.3%)	
Race	Non-White	34 (50%)	39 (48.8%)	0.879
	White	34 (50%)	41 (51.2%)	
Non-White Race Breakdown	American Indian or Alaska Native	1 (1.5%)	0 (0%)	
	Asian	2 (2.9%)	1 (1.3%)	
	Black or African American	29 (42.6%)	37 (46.3%)	
	Other	2 (2.9%)	1 (1.3%)	
BMI (kg/m^2^)	Non-obese (<30)	49 (72.6%)	58 (72.5%)	0.952
	Obese (≥30)	19 (27.4%)	22 (27.5%)	
	Median (IQR)	25.8 (23.4–30.9)	26.7 (24.9–30.6)	0.325
Cancer Status	Survivorship	38 (55.9%)	50 (62.5%)	0.414
	Active Treatment	30 (44.1%)	30 (37.5%)	
Charlson Comorbidity Index	Severe	40 (58.8%)	42 (52.5%)	0.441
	Non-severe	28 (41.2%)	38 (47.5%)	
	Median (IQR)	4 (3–6)	4 (4–6)	0.115
ECOG Performance Status	ECOG 0	47 (69.1%)	60 (75%)	0.426
	ECOG 1+	21 (30.9%)	20 (25%)	
	Median (IQR)	0 (0–1)	0 (0.0–0.5)	0.375
Physical Function Domain *	Normal	30 (44.8%)	31 (40.8%)	
	Mild	20 (29.9%)	24 (31.6%)	0.889
	Moderate/Severe	17 (25.3%)	21 (27.6%)	
	Median (IQR)	43.2 (39.9–56.9)	43.4 (38.7–47.75)	0.744
Anxiety Domain *	Normal	40 (59.7%)	49 (64.5%)	
	Mild	16 (23.9%)	13 (17.1%)	0.601
	Moderate/Severe	11 (16.4%)	14 (18.4%)	
	Median (IQR)	52.1 (40.3–57.8)	50.3 (40.3–57.8)	0.323
Depression Domain *	Normal	47 (70.1%)	59 (77.6%)	
	Mild	15 (22.4%)	10 (13.2%)	0.344
	Moderate/Severe	5 (7.5%)	7 (9.2%)	
	Median (IQR)	48.9 (41.0–55.9)	48.9 (41.0–54.2)	0.5
Fatigue Domain *	Normal	39 (58.2%)	44 (57.8%)	
	Mild	12 (17.9%)	16 (21.1%)	0.858
	Moderate/Severe	16 (23.9%)	16 (21.1%)	
	Median (IQR)	51 (46.4–59.2)	51 (46.2–57.9)	0.778
Sleep Disturbance Domain *	Normal	39 (58.2%)	60 (78.9%)	
	Mild	19 (28.4%)	4 (5.3%)	<0.001
	Moderate/Severe	9 (13.4%)	12 (15.8%)	
	Median (IQR)	53 (47.7–57.5)	51.1 (46.4–54.5)	0.051
Social Participation Domain *	Normal	39 (58.2%)	47 (61.8%)	
	Mild	21 (31.3%)	17 (22.4%)	0.382
	Moderate/Severe	7 (10.4%)	12 (15.8%)	
	Median (IQR)	48.2 (44.2–53.5)	50 (44.2–55.0)	0.823
Pain Interference Domain *	Normal	31 (46.3%)	34 (44.7%)	
	Mild	17 (25.3%)	20 (26.2%)	0.983
	Moderate/Severe	19 (28.4%)	22 (28.9%)	
	Median (IQR)	55.7 (41.6–60.3)	55.7 (49.4–60.8)	0.563

* 143 of 148 patients completed a baseline PROMIS-29 questionnaire. Abbreviations: Interquartile range (IQR).

**Table 3:** Study cohort characteristics associated with yoga attendance.

Covariate	Subgroup	IRR (95% CI)	*p*-Value
Gender	Female	0.78 (0.46–1.32)	0.347
	Male	Reference	
Race	Non-White	0.94 (0.59–1.48)	0.79
	White	Reference	
Age	**≥60**	**1.74 (1.05–2.90)**	**0.033**
	**<60**	**Reference**	
BMI (kg/m^2^)	Obese (≥30 BMI)	1.04 (0.63–1.72)	0.817
	Non-obese (<30 BMI)	Reference	
Cancer Status	Active Treatment	1.09 (0.66–1.81)	0.712
	Survivorship	Reference	
ECOG	1+	0.78 (0.44–1.36)	0.385
	0	Reference	
CCI	Severe (≥5 CCI)	1.00 (0.61–1.63)	0.994
	Non-Severe (<5 CCI)	Reference	

Statistically significant (*p* < 0.05) results are bolded. Abbreviations: Eastern Cooperative Oncology Group (ECOG); Body Mass Index (BMI); Chalson comorbidity index (CCI).

**Table 4:** PROMIS-29 domains associated with yoga attendance.

Covariate	Subgroup	IRR (95% CI)	*p*-Value
Physical Function Domain	Moderate/Severe	1.11 (0.52–2.36)	0.771
	Mild	1.07 (0.58–1.94)	0.824
	Normal	Reference	
Anxiety Domain	Moderate/Severe	1.25 (0.47–3.32)	0.648
	Mild	0.84 (0.40–1.75)	0.654
	Normal	Reference	
Depression Domain	Moderate/Severe	0.98 (0.30–3.16)	0.985
	Mild	0.67 (0.29–1.56)	0.358
	Normal	Reference	
Fatigue Domain	Moderate/Severe	0.99 (0.43–2.27)	0.986
	Mild	1.30 (0.69–2.46)	0.413
	Normal	Reference	
Sleep Disturbance Domain	Moderate/Severe	0.83 (0.38–1.83)	0.652
	Mild	0.26 (0.09–0.74)	**0.012**
	Normal	Reference	
Social Participation Domain	Moderate/Severe	0.97 (0.36–2.64)	0.965
	Mild	0.79 (0.38–1.65)	0.542
	Normal	Reference	
Pain Interference Domain	Moderate/Severe	1.20 (0.62–2.40)	0.581
	Mild	1.15 (0.64–2.08)	0.623
	Normal	Reference	

Statistically significant (*p* < 0.05) results are bolded. Abbreviations: Incidence rate ratio (IRR); 95% Confidence interval (95%CI).

**Table 5:** Pre and post-yoga PROMIS-29 domain score distributions.

Covariate	Subgroup	Pre-Yoga (N = 62; 95% CI)	Post-Yoga (N = 62; 95% CI)	*p*-Value
Physical Function Domain	Normal	25 (40.3%; 28.1–53.6%)	30 (48.4%; 35.5–61.4%)	
	Mild	21 (33.9%; 22.3–47.0%)	14 (22.6%; 12.9–35.0%)	
	Moderate/ Severe	16 (25.8%; 15.5–38.5%)	18 (29.0%; 18.1–41.9%)	
	Median (IQR)	43.4 (38.7–47.8)	43.5 (39.0–56.9)	0.644
Anxiety Domain	Normal	41 (66.1%; 52.9–77.7%)	41 (66.1%; 53.0–77.7%)	
	Mild	10 (16.1%; 8.1–27.7%)	11 (17.7%; 9.2–29.5%)	
	Moderate/ Severe	11 (17.8%; 9.2–29.5%)	10 (16.2%; 8.1–27.7%)	
	Median (IQR)	50.3 (40.3–57.5)	48.9 (40.3–57.8)	0.603
Depression Domain	Normal	50 (80.6%; 68.3–89.6%)	52 (83.9%; 72.3–92.0%)	
	Mild	7 (11.3%; 4.6–21.9%)	6 (9.7%; 3.6–19.8%)	
	Moderate/ Severe	5 (8.1%; 2.7–17.8%)	4 (6.4%; 1.8–15.7%)	
	Median (IQR)	48.9 (41.0–52.2)	48.9 (41.0–52.0)	0.467
Fatigue Domain	Normal	37 (59.7%; 46.4–71.9%)	42 (67.7%; 54.7–79.1%)	
	Mild	13 (21.0%; 11.7–33.2%)	8 (12.9%; 5.7–23.9%)	
	Moderate/ Severe	12 (19.3%; 10.4–31.4%)	12 (19.4%; 10.4–31.4%)	
	Median (IQR)	51 (46.2–57.1)	48.9 (48.6–57.1)	0.9
Sleep Disturbance Domain	Normal	50 (80.6%; 68.3–89.6%)	48 (77.4%; 65.0–87.1%)	
	Mild	3 (4.8%; 1.0–13.5%)	7 (11.3%; 4.6–21.9%)	
	Moderate/ Severe	9 (15.6%; 6.9–25.8%)	7 (11.3%; 4.6–21.9%)	
	Median (IQR)	49.6 (46.4–54.2)	51.3 (46.4–54.5)	0.442
Social Participation Domain	Normal	39 (62.9%; 49.7–74.8%)	41 (66.1%; 53.0–77.7%)	
	Mild	13 (21.0%; 11.7–33.2%)	13 (21.0%; 11.7–33.2%)	
	Moderate/ Severe	10 (16.1%; 8.1–27.7%)	8 (12.9%; 5.7–23.9%)	
	Median I(QR)	50.1 (44.2–55.9)	51.8 (44.2–55.6)	0.242
Pain Interference Domain	Normal	28 (45.2%; 32.5–58.3%)	26 (41.9%; 29.5–55.2%)	
	Mild	18 (29.0%; 18.1–41.9%)	22 (35.5%; 23.7–48.7%)	
	Moderate/ Severe	16 (25.8%; 15.5–38.5%)	14 (22.6%; 12.9–35.0%)	
	Median (IQR)	55.7 (41.6–60.0)	55.7 (52.2–58.9)	0.371

Abbreviations: Interquartile range (IQR).

## Data Availability

The original contributions presented in this study are included in the Supplementary Material. Further inquiries can be directed to the corresponding author.

## Supplementary Materials

The following supporting information can be downloaded at: https://www.mdpi.com/article/10.3390/healthcare14213830/s1. Supplemental File S1: Patient survey developed for the purposes of this study to assess yoga program perceptions. Supplemental Table S1: Cohort Distribution of Primary Cancer Diagnosis.
