# Supplementary material for: Facility-Based Yoga for Adult Cancer Patients and Survivors at a National Cancer Institute (NCI)-Designated Comprehensive Cancer Center: A Retrospective Study of Program Utilization and Barriers to Care
Source: Healthcare (Basel). 2026 Nov 9;14(21):3830. doi: 10.3390/healthcare14213830 (PMC13763535; doi:10.3390/healthcare14213830)
Supplement: Supplementary file 1 [file healthcare-14-03830-s001.zip › Supplemental File S1.pdf]

Supplementary File S1. Patient survey developed for the purposes of this study to assess yoga program perceptions.

Oncology Yoga Satisfaction Survey

Winship Integrative Oncology & Survivorship

Please answer the following questions as it relates to your experience in Integrative Oncology Yoga. Your feedback is valued and we look forward to hearing from you. Please mark only 1 answer per question.

1) Friendliness/courtesy of the yoga teacher

- ☐ 1- Very Poor
- ☐ 2- Poor
- ☐ 3- Fair
- ☐ 4- Good
- ☐ 5- Very Good
- ☐ N/A

2) Concern the yoga teacher showed for your questions or worries

- ☐ 1- Very Poor
- ☐ 2- Poor
- ☐ 3- Fair
- ☐ 4- Good
- ☐ 5- Very Good

3) Degree to which the yoga teacher talked with you using words you could understand

- ☐ 1- Very Poor
- ☐ 2- Poor
- ☐ 3- Fair
- ☐ 4- Good
- ☐ 5- Very Good
- ☐ N/A

4) Amount of time the yoga teacher spent with you

- ☐ 1- Very Poor
- ☐ 2- Poor
- ☐ 3- Fair
- ☐ 4- Good
- ☐ 5- Very Good

5) Your confidence in this yoga teacher

- ☐ 1- Very Poor
- ☐ 2- Poor
- ☐ 3- Fair
- ☐ 4- Good
- ☐ 5- Very Good
- ☐ N/A

6) Likelihood of your recommending this yoga teacher to others

- ☐ 1- Very Poor
- ☐ 2- Poor
- ☐ 3- Fair
- ☐ 4- Good
- ☐ 5- Very Good
- ☐ N/A

7) Comments (describe good or bad experiences)
