# Supplementary material for: Facility-Based Yoga for Adult Cancer Patients and Survivors at a National Cancer Institute (NCI)-Designated Comprehensive Cancer Center: A Retrospective Study of Program Utilization and Barriers to Care
Source: Healthcare (Basel). 2026 Nov 9;14(21):3830. doi: 10.3390/healthcare14213830 (PMC13763535; doi:10.3390/healthcare14213830)
Supplement: Supplementary file 1 [file healthcare-14-03830-s001.zip › Supplemental Table S1.pdf]

Supplemental Table S1. Cohort Distribution of Primary Cancer Diagnosis

**Primary Diagnosis**	**Total (N=148)***
Brain Cancer	1 (0.7%)
Breast Cancer	87 (58.8%)
Cervical Cancer	2 (1.6%)
Colorectal Cancer	5 (3.4%)
Endometrial Cancer	2 (1.6%)
Head and Neck Cancer	5 (3.4%)
Kidney Cancer	3 (2%)
Lung Cancer	6 (4.1%)
Lymphoma	2 (1.6%)
Multiple Myeloma	9 (6.1%)
Ovarian Cancer	4 (2.7%)
Pancreatic Cancer	2 (1.6%)
Prostate Cancer	13 (8.8%)
Sarcoma	3 (2%)
Stomach Cancer	1 (0.7%)
Thyroid Cancer	1 (0.7%)
Uterine Cancer	2 (1.6%)

***Values represented as *n* (%).**
